# 

**Published:** 1849-01

**Authors:** 


### CONTENTS OF THE DENTAL REGISTER OF THE WEST.

JANUARY, 1849.

Art. 1. Clergymen and Physicians as Dentists, by Dr. J. W. Power ........ Page 53
" 2. Letter from Professor Powell ........ 58
" 3. Voluntary Associations and Professional Reform, by Henry James Brown, A. M., M. D. ........ 61
" 4. A case of Hæmorrhage after the removal of a Canine Root, by J. A. Weaver, D. D. S. ........ 66
" 5. From my Note Book, No. 1, by Dr. B. T. Whitney ........ 68
" 6. Third Dentition ........ 70
" 7. Letter from Dr. B. T. Whitney ........ 75
" 8. The Importance of a Correct Medico-Dental Education, by J. S. King, D. D. S. ........ 77
" 9. Strictures, by Wm. A. Pease, Dentist ........ 83
" 10. Anchylosis of the Jaw ........ 89
" 11. The Lamp, by Dr. Taft ........ 94
" 12. Remarks on Du Bouchet's Nostrum, by A. Berry, D.D.S. ........ 96
" 13. A case of Acute Inflammation of the Sublingual Glands, by Edward M. Boykin, M. D. ........ 97
" 14. Latest Humbug ........ 100
" 15. Lawrence's Improved Portable Blow-Pipe ........ 103
" 16. Composition of a Salivary Calculus, taken from a Horse ........ 104
" 17. Extract of a Letter from Dr. Peebles ........ 105
" 18. Notice to Manufacturers ........ 105

EDITORIAL.

" 19. Cholera ........ 106
" 20. American Journal of Dental Science ........ 107
" 21. Agents for the Register ........ 107
" 22. Contents—Errata ........ 108

